# Application of green first derivative synchronous spectrofluorometric method for quantitative analysis of fexofenadine hydrochloride and pseudoephedrine hydrochloride in pharmaceutical preparation and spiked human plasma

**DOI:** 10.1186/s13065-022-00855-5

**Published:** 2022-08-19

**Authors:** Sherif Ramzy, Ahmed H. Abdelazim, Mohamed A. Hasan

**Affiliations:** Pharmaceutical Analytical Chemistry Department, Faculty of Pharmacy, Al-Azhar University, 11751 Nasr City, Cairo, Egypt

**Keywords:** Fexofenadine hydrochloride, Pseudoephedrine hydrochloride, Synchronous fluorescence, Green chemistry

## Abstract

Fexofenadine hydrochloride and pseudoephedrine hydrochloride are prescribed in a combined dosage form for the treatment of allergic rhinitis. In the present work, a sensitive synchronous fluorescence spectroscopic method was applied in conjunction with first derivative for quantitative estimation of fexofenadine hydrochloride and pseudoephedrine hydrochloride in pure form, pharmaceutical tablets and spiked human plasma. Fexofenadine hydrochloride showed its conventional emission spectrum at 294 nm when excited at 267 nm. On the other hand, pseudoephedrine hydrochloride showed its conventional emission spectra at 286 nm when excited at 261 nm. The fluorescence intensities were greatly enhanced by the use of sodium dodecyl sulphate as a micellar surfactant. Application of the synchronous mode to measure the fluorescence spectra of the above drugs provided sharp narrowing bands, but the overlap was not completely resolved. Derivatization of the synchronous spectra to the first order completely resolved the overlap of the fluorescence spectra and allowed simultaneous quantitative determination of the drugs under study. Fexofenadine hydrochloride and pseudoephedrine hydrochloride could be determined from their first-order synchronous spectra at 286 and 294 nm, respectively, without interfering with each other. The method showed linearity with an excellent correlation coefficient in the concentration range of 100–1500 ng/mL for Fexofenadine hydrochloride and 50–1000 ng/mL for pseudoephedrine hydrochloride. The method was successfully applied for the simultaneous determination of the studied drugs in pharmaceutical formulation, with mean percent recoveries for Fexofenadine hydrochloride and pseudoephedrine hydrochloride of 99.49 ± 0.931 and 98.67 ± 0.634, respectively, and in spiked human plasma, with mean percent recoveries for Fexofenadine hydrochloride and pseudoephedrine hydrochloride of 95.21 ± 1.938 and 94.89 ± 1.763, respectively. Furthermore, the greenness of the described method was assessed using four different tools namely, the national environmental method index, the analytical eco-scale, the green analytical procedure index and the AGREE evaluation method. The proposed method seemed to be superior to the reported HPLC method with respect to the metrics of the greenness characters.

## Introduction

Fluorescence spectrometry is an important analytical tool for drug quantification due to its high sensitivity and low cost [1]. However, its application for simultaneous and direct quantification of mixtures of multiple drugs is limited due to spectral overlap problems [2, 3]. These overlap problems could be solved by applying the synchronous fluorescence detection mode, in which both excitation and emission monochromators are scanned simultaneously, resulting in simplification and narrowing of spectral bands. In addition, derivatization of such synchronous to first- or second-order spectra provides synergistic effects to improve selectivity and resolution [4–6].

**Fig. 1:**
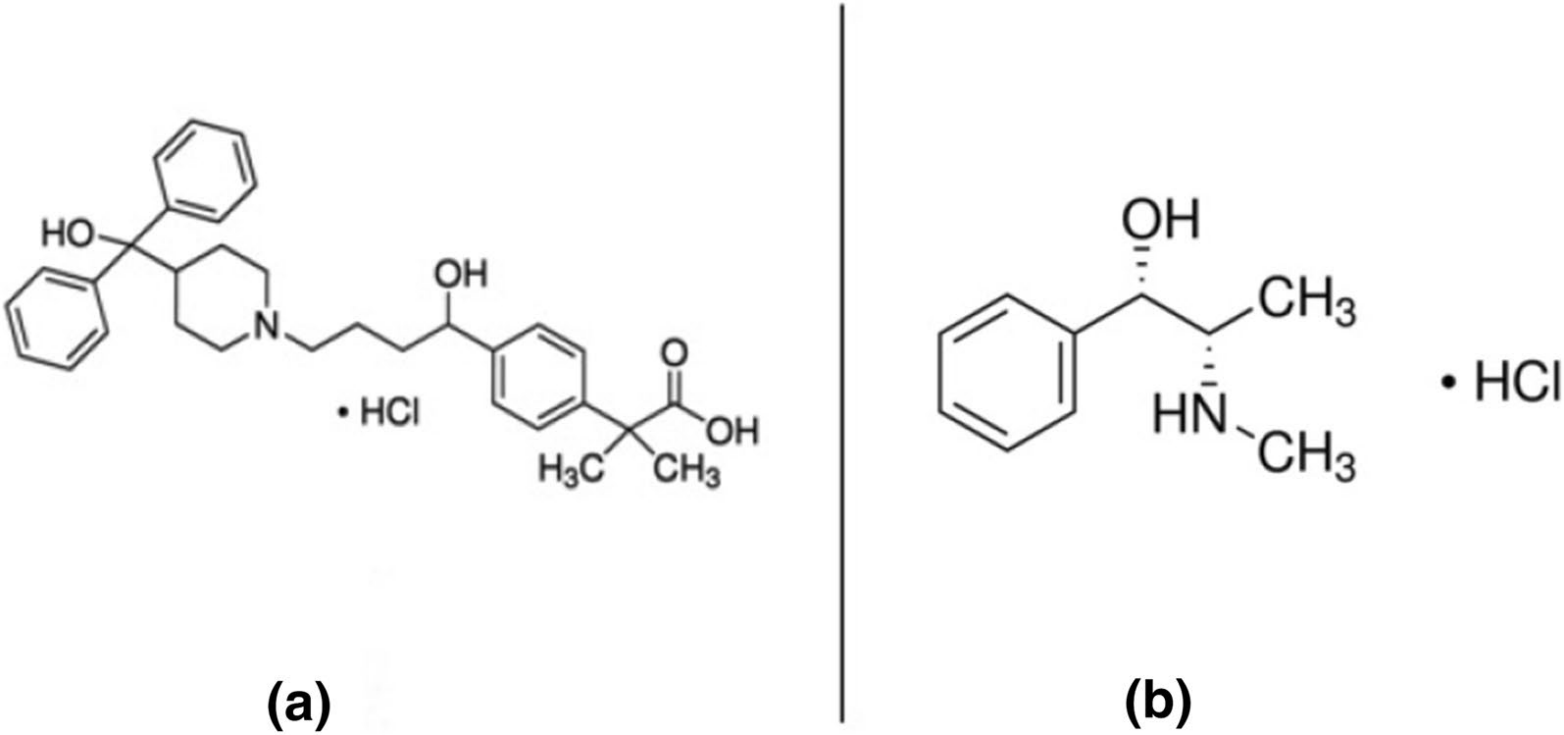
Structural formula of FEX (**a**) and PSE (**b**)

Telfast-D® is a combination tablet designed to relieve symptoms of seasonal allergic rhinitis by providing an immediate release of fexofenadine hydrochloride equivalent to 60 mg and an extended release of pseudoephedrine hydrochloride equivalent to 120 mg. Fexofenadine hydrochloride (FEX), Fig. 1a, is an antihistamine that selectively antagonizes peripheral H_1_ receptor activity. Pseudoephedrine hydrochloride (PSE), shown in Fig. 1b, is a decongestant and agonist of the α-adrenergic receptor [7]. Few techniques have been published for simultaneous quantitative analysis of FEX and PSE, including HPLC [8, 9], LC–MS-MS [10], and spectroscopy [11]. As it turned out, no published article simultaneously tested the fluorescence behavior of FEX and PSE in the combination tablet. In this article, the authors developed the first green spectrofluorometric method for the simultaneous determination of FEX and PSE. Initial tests of the recorded fluorescence spectra of the investigated drugs show strong overlap with relatively low sensitivity. To overcome these difficulties, sodium dodecyl sulphate was used as a micellar system to increase the spectral sensitivity. The synchronous first derivative spectrofluorometric method was then applied to resolve the overlapping spectra and selectively quantify the drugs under study. Additionally, the greenness of the described method was evaluated on a scientific basis using the national environmental method index [12], the analytical eco-scale [13], the green analytical procedure index [14], and the AGREE evaluation method [15], in order to eliminate the use of toxic chemicals and solvents and be eco-friendly. The developed method was successfully applied for the simultaneous quantification of FEX and PSE, both in pure form, spiked human plasma, and tablet formulation, with higher sensitivity and with minimum impact on the environment.

## Methods

### ***Materials, chemicals and solvents***

FEX and PSE high purity raw materials were supplied by Benchmark Health Company, Cairo, Egypt. Telfast-D^®^ tablets, a combination of FEX 60 mg and PSE 120 mg, were obtained from the pharmacy. Chemicals of high purity analytical grades, solvents of HPLC grades, and double distilled water, were used throughout the analysis procedures. Methyl cellulose (MC, 0.5% aqueous solution), Tween 80 (0.5% aqueous solution), sodium dodecyl sulphate (SDS, 0.5% aqueous solution), boric acid, glacial acetic acid, potassium biphthalate, potassium chloride, sodium hydroxide (0.1 N aqueous solution), hydrochloric acid (0.1 N aqueous solution), and sodium acetate, were from El Nasr Company, Abu Zaabal, Egypt. β-Cyclodextrin (β- CD, 0.5% aqueous solution) was from Fluka Chemie, Germany. Cetyltrimethylammonium bromide (CTAB, 0.5% aqueous solution) was from Winlab, Welwyn, UK. Acetonitrile, chloroform, dichloromethane, ethanol, methanol, and tetrahydrofuran, were from Sigma‐Aldrich, Germany. Drug-free human plasma of healthy volunteers was supplied by Blood Bank, Al-Azhar University Hospital, Damietta, Egypt. Buffers for different pH ranges were prepared according to the US pharmacopeia regulations [16].

### Instrumentation

Jasco FP-6200 Spectrofluorometer was used for all measurements (Tokyo, Japan). Jasco Spectra Manager™ software was used to manipulate the measured spectra. pH meter; Jenway 3510 (Staffordshire, England) and Rotary evaporator; Scilogex-RE 100-pro (Rocky Hill, USA) were also used.

### Standard solutions

FEX and PSE standard solution (100 μg/mL) were prepared in ethanol. Further dilution with ethanol was performed to obtain a working solution (10 μg/mL) for each drug.

### Procedures

#### Linearity and calibration graphs

Serial diluted solutions of FEX and PSE were prepared by pipetting into two separate sets of 10-mL volumetric flasks containing (1–15 µg) and (0.5–10 µg) FEX and PSE, respectively, together with 1.5 mL of HCl buffer (pH 2) and 2 mL of 0.5% aqueous solution SDS. The volume of each flask was adjusted to the mark with ethanol. The solution of each flask was scanned in the spectrofluorometer in synchronous mode at delta lambda = 25 nm. A blank experiment was performed simultaneously. The recorded synchronous spectra were transformed to the corresponding first-order derivative with data points = 5. The peak amplitudes of the first-derivative spectra were measured at 286 nm and 294 nm for FEX and PSE, respectively. The measured values were plotted against the respective drug concentrations in ng/mL to obtain the calibration graphs, and the corresponding regression equations were derived. In general, all described procedures were performed in accordance with relevant guidelines.

### Analysis of synthetic mixtures

Various synthetic mixtures were prepared containing different concentrations of FEX and PSE in a ratio corresponding to the pharmaceutical tablets (1:2). The mixtures were analyzed according to the procedure given under the linearity and calibration diagrams.

### Application to spiked human plasma

Aliquots of different concentrations in a ratio corresponding to the pharmaceutical tablet were transferred from the working standard solutions of FEX and PSE to a series of 10-mL centrifugation tubes. To each tube, 1 mL of the human plasma samples was added, followed by 3 mL of acetonitrile. The tubes were shaken on a vortex mixer for 1 min and then centrifuged for 30 min. The resulting supernatants were evaporated to dryness in a rotary evaporator. The residues were dissolved in an appropriate volume of ethanol and then transferred to a series of 10-mL volumetric flasks and made up to volume with ethanol. Blank samples were prepared under similar conditions. The prepared samples were analyzed according to the procedure given under the linearity and calibration diagrams.

### Application to pharmaceutical tablets

Ten Telfast-D^®^ tablets (FEX 60 mg and PSE 120 mg per tablet) were weighed and then ground to a fine powder. A weighed powder equivalent to one tablet was accurately transferred to a 100-mL volumetric flask containing 50 mL ethanol, shaken vigorously for 20 min, filtered, and adjusted to 100 mL with ethanol. The recommended five samples were prepared by further dilution with ethanol and analyzed quantitatively according to the procedure given under the linearity and calibration charts.

## Results and discussion

### Spectral characteristics

**Fig.2:**
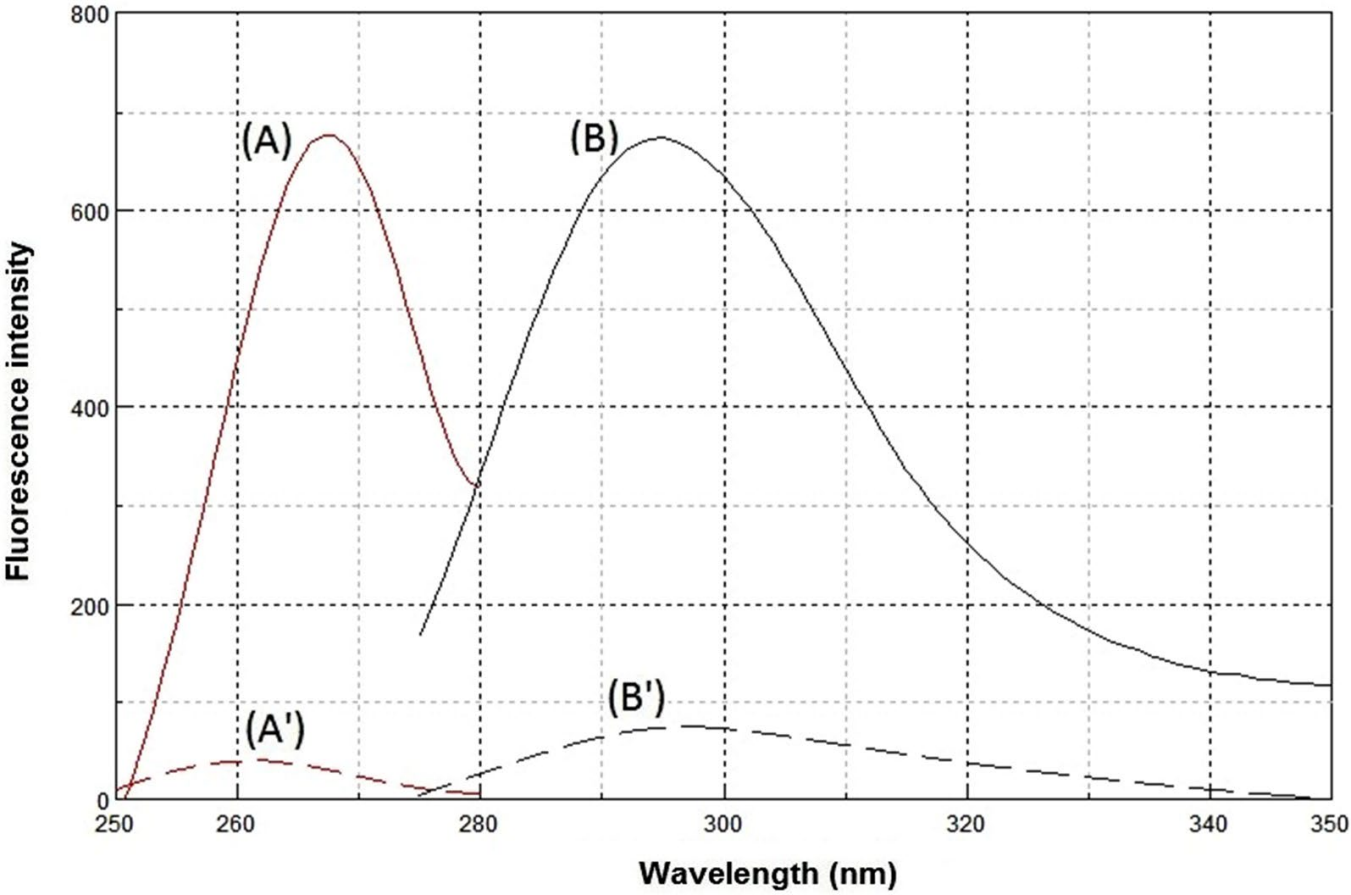
Excitation (**A**) and emission (**B**) spectra of FEX (1000 ng/ml) in ethanol

**Fig.3:**
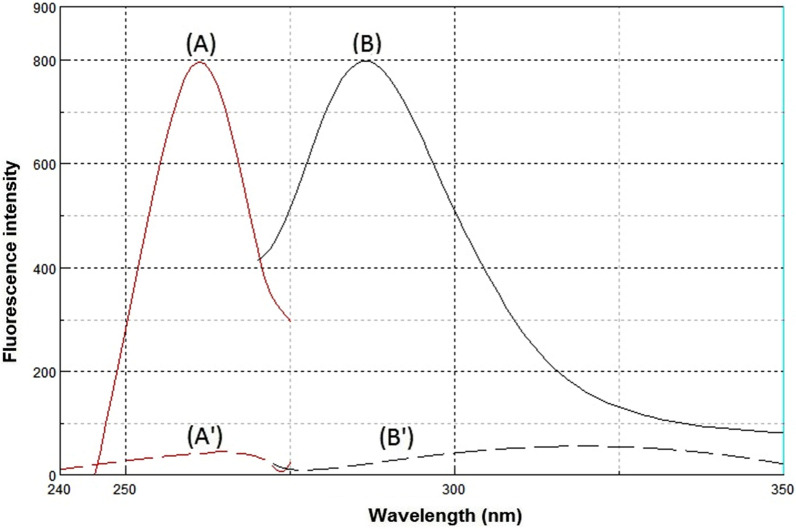
Excitation (**A**) and emission (**B**) spectra of PSE (800 ng/ml) in ethanol

**Fig.4:**
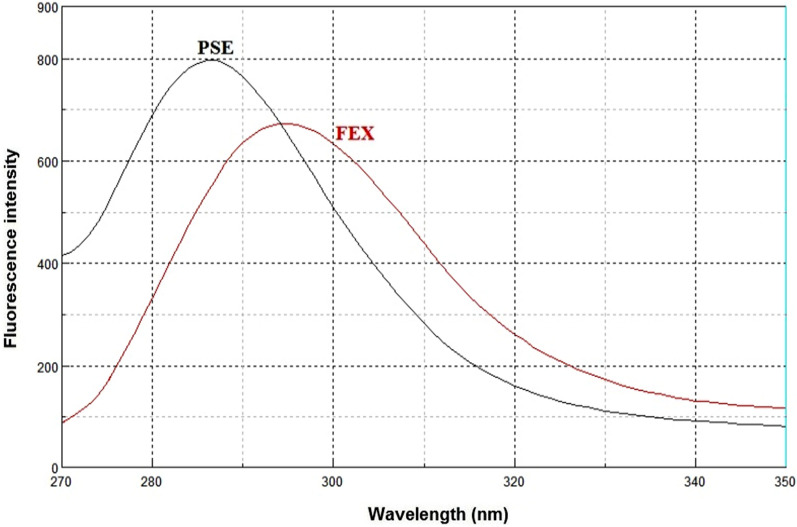
Emission spectra of FEX (1000 ng/ml) and PSE (800 ng/ml) in ethanol after excitation at λex. = 265 nm

**Fig.5:**
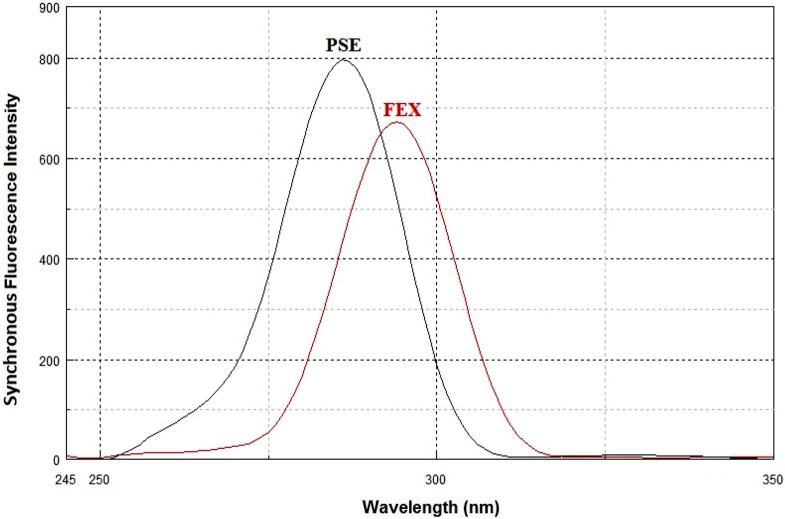
Synchronous fluorescence spectra of FEX (1000 ng/ml) and PSE (800 ng/ml) in ethanol using Δλ = 25 nm

**Fig.6:**
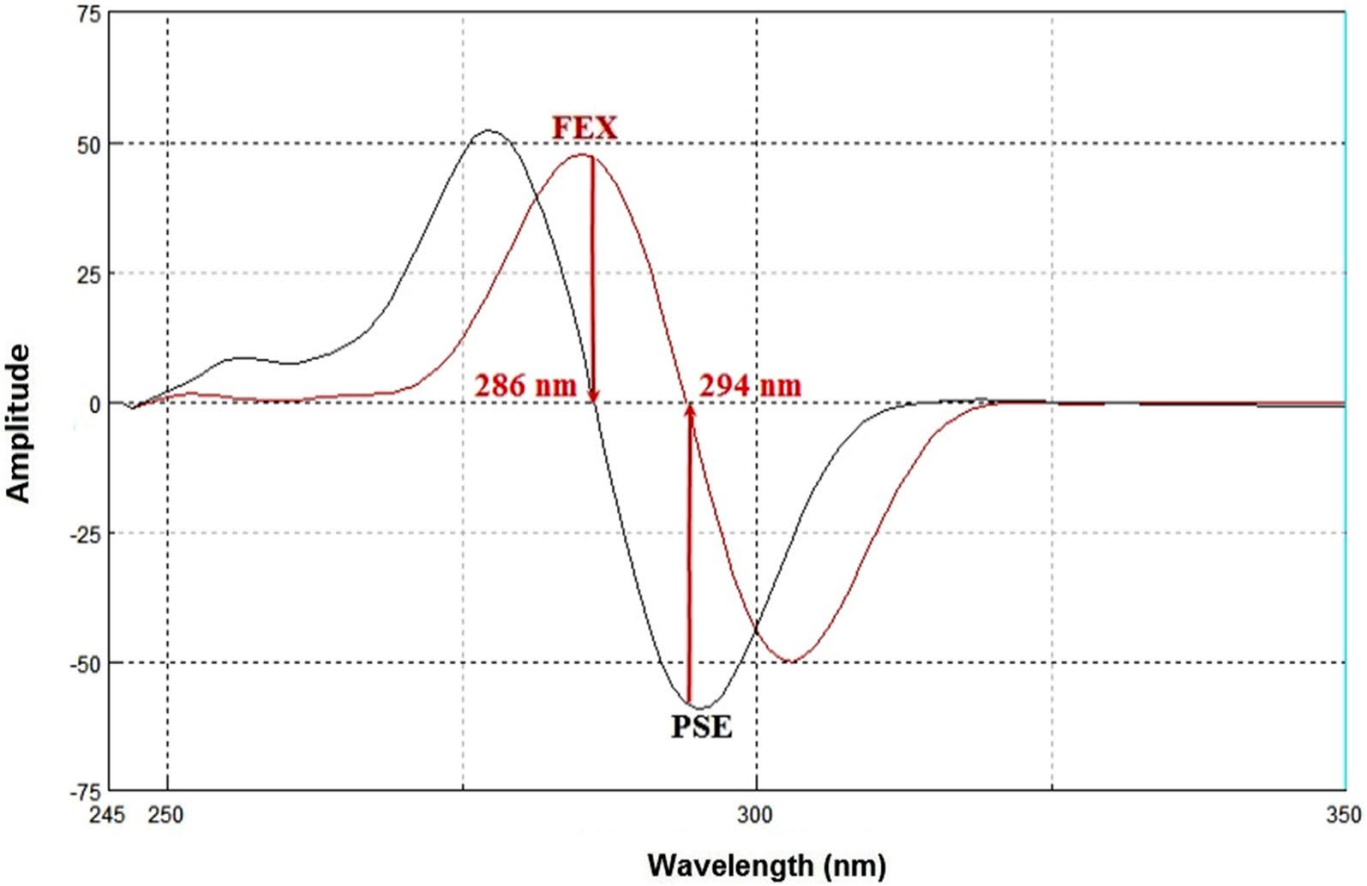
First derivative synchronous fluorescence spectra of FEX (1000 ng/ml) and PSE (800 ng/ml) in ethanol using Δλ = 25 nm

Synchronous fluorescence spectroscopy plays an important role in resolving the conventional overlapping spectra of a multicomponent by converting the broader spectra into sharper and narrower spectra. In general, the resolution and selectivity produced by the synchronous fluorescence mode are not sufficient to fully resolve the overlap of multicomponent. Therefore, the combination of the derivative technique with synchronous fluorescence spectroscopy was recommended. FEX and PSE have native emission fluorescence spectra at 294 nm and 286 nm after excitation at 267 nm and 261 nm, respectively. The fluorescence intensities were greatly enhanced by using SDS as a micellar surfactant (Figs. 2, 3). The recorded emission fluorescence spectra of FEX and PSE overlapped greatly, making their direct determination in the conventional fluorescence mode difficult (Fig. 4). Application of the synchronous mode to measure the fluorescence spectra of FEX and PSE provided sharp narrowing bands, but the overlap was not completely resolved, Fig. 5. Derivatization of such synchronous spectra to the first order completely resolved the spectral overlap and allowed simultaneous determination of the drugs under study. FEX and PSE could be determined from their first-order synchronic spectra at 286 and 294 nm, respectively, without interfering with each other (Fig. 6).

### Method optimization

The factors affecting the sensitivity and selectivity of the synchronous fluorescence spectra of FEX and PSE were investigated and optimized. The factors studied were the selection of the optimal Δλ, the effect of the diluting solvent, the type and amount of surfactant, the pH effect, and the buffer volume. During these studies, fixed amounts of the studied drugs were used and each factor was changed separately while the others were kept constant.

**Fig.7:**
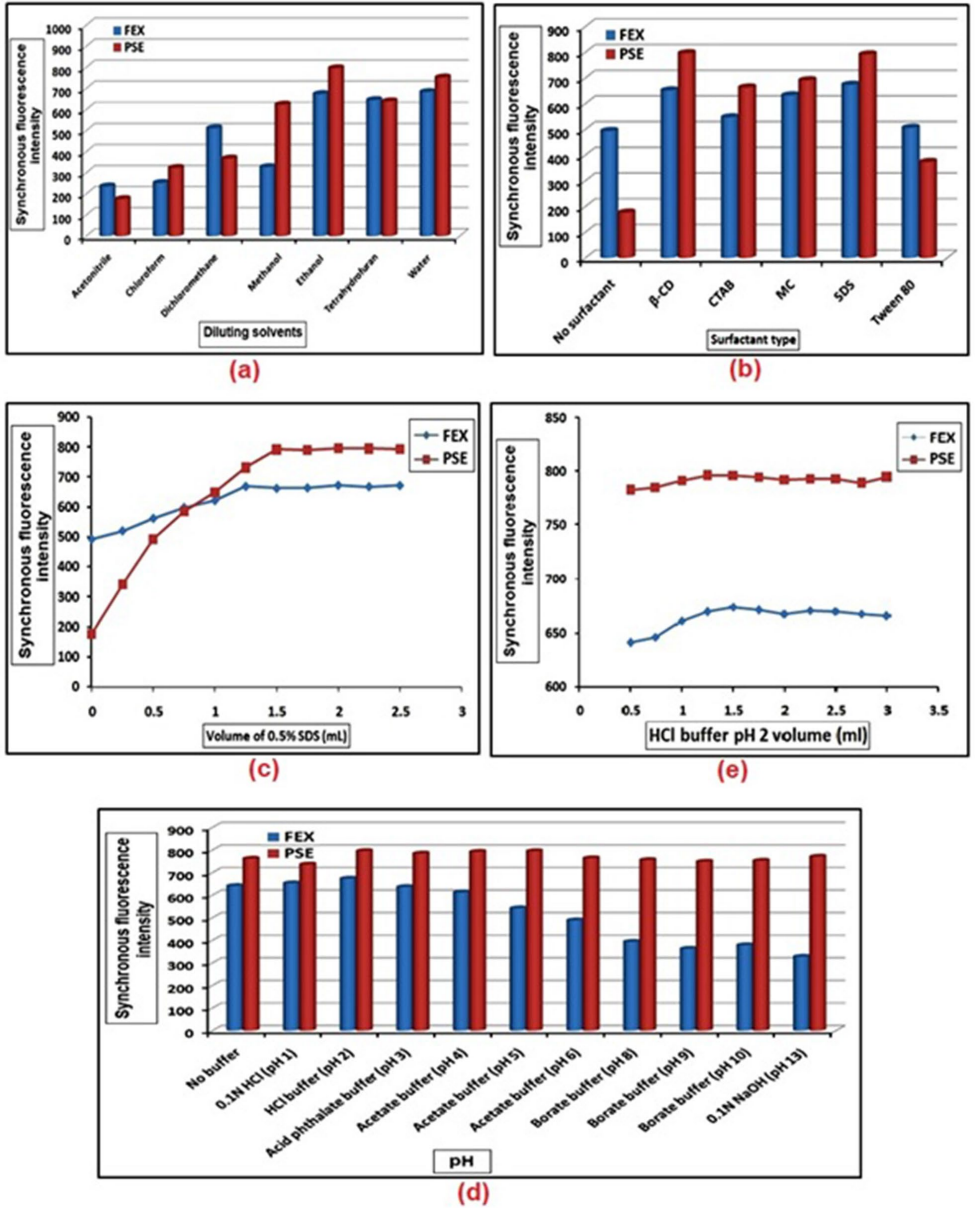
Optimization of experimental conditions for FEX and PSE including diluting solvent (**a**), surfactant type (**b**), surfactant volume (**c**), buffer type (**d**) and buffer volume (**e**)

Synchronous fluorescence spectra of FEX and PSE recorded over a wide range of Δλ-values (20–100 nm) were compared for highest sensitivity and better resolution. Two narrowing peaks of FEX and PSE with good resolution and highest signal values were obtained by scanning at Δλ = 25. On the other hand, after recording the synchronous fluorescence spectra of FEX and PSE in various solvents, including water, acetonitrile, chloroform, dichloromethane, ethanol, methanol, and tetrahydrofuran, it was found that ethanol gave the highest spectral intensity of the two agents (Fig. 7a). Different surfactants such as MC, Tween 80, CTAB, SDS and β- CD were tried to increase the sensitivity of the studied drugs. The result showed that SDS with a volume of 2 mL greatly increased the synchronous fluorescence intensity of FEX and PSE, Figs. 7b and c. In addition, the effect of pH on the synchronous fluorescence intensity of FEX and PSE was investigated by adding different buffers over the whole pH range. The result showed that the highest synchronous fluorescence intensity of FEX and PSE was obtained by adding HCl buffer (pH 2) with a volume of 1.5 mL (Fig. 7d and e).

### Method validation

The method used was validated in accordance with the guidelines of ICH [17]. The data on accuracy, repeatability and mean precision, limits of detection (LOD) and quantification (LOQ), robustness, linearity and regression trend line are presented in Table 1. The selectivity of the applied method for the simultaneous determination of the investigated drugs in their synthetic mixtures was verified and confirmed, as shown in Table 2. The selectivity of the applied method was also checked and confirmed by the successful quantification of the studied drugs without any interference by tablet excipients (using the standard addition technique) (Table 3) or by endogenous components of human plasma (Table 4).

**Table 1:** Regression and validation data for the determination of FEX and PSE by the proposed method

Parameters	FEX	PSE
Wavelength (nm)	286	294
Linearity range (ng/mL)	100–1500	50–1000
LOD (ng/mL)	24.15	12.88
LOQ (ng/mL)	73.49	39.21
Slope	0.0377	0.0623
Intercept	9.6551	8.8644
Coefficient of determination (r^2^)	0.9995	0.9994
Accuracy (%R)^a^	99.13	98.46
Precision (RSD)^b^		
Repeatability	1.369	0.493
Intermediate precision	1.012	1.207
Robustness (%R ± RSD)		
Δλ (± 1 nm.)	100.63 ± 0.926	99.20 ± 1.017
pH (± 0.1)	99.98 ± 1.066	99.82 ± 1.314
Buffer volume (± 0.1 mL)	100.89 ± 0.878	100.12 ± 0.950

^a^Average of nine determinations (three concentrations repeated three times)

^b^RSD of nine determinations (three concentrations repeated three times)

**Table 2:** Determination of FEX and PSE in synthetic laboratory mixtures by the proposed method

FEX			PSE		
Added (ng/mL)	Found^a^ (ng/mL)	%Recovery	Added (ng/mL)	Found^a^ (ng/mL)	%Recovery
100	100.94	100.94	200	196.7	98.35
150	152.75	101.83	300	300.57	100.19
200	203.44	101.72	400	397.64	99.41
250	249.65	99.86	500	491.9	98.38
300	302.73	100.91	600	601.56	100.26
Mean ± RSD		101.05 ± 0.783	Mean ± RSD		99.32 ± 0.938

^a^Average of three determinations

**Table 3:** Recovery study of FEX and PSE by standard addition technique using the proposed method

Drug	Pharmaceutical taken (ng/mL)	Pharmaceutical found^a^ (ng/mL)	Pure added (ng/mL)	Pure found^b^ (ng/mL)	% Recovery
FEX	150	149.24	100	99.27	99.27
			200	196.62	98.31
			300	303.24	101.08
	Mean ± %RSD				99.55 ± 1.431
PSE	300	296.01	100	100.12	100.12
			200	196.38	98.19
			300	297.21	99.07
	Mean ± %RSD				99.13 ± 0.975

^a^ Average of five determinations

^b^ Average of three determinations

**Table 4:** Determination of FEX and PSE in spiked human plasma by the proposed method

FEX			PSE		
Added (ng/mL)	Found^a^ (ng/mL)	%Recovery	Added (ng/mL)	Found^a^ (ng/mL)	%Recovery
100	95.38	95.38	200	184.48	92.24
150	139.79	93.19	300	288.03	96.01
200	192.54	96.27	400	377.28	94.32
250	233.98	93.59	500	477.35	95.47
300	292.83	97.61	600	578.34	96.39
Mean ± RSD		95.21 ± 1.938	Mean ± RSD		94.89 ± 1.763

^a^ Average of three determinations

### Application to spiked human plasma

The developed method was successfully used for the quantification of FEX & PSE in spiked human plasma due to its low detection limit. The mean plasma C_max_ for FEX and PSE, based on population pharmacokinetic analysis, was 191 ng/mL and 206 ng/mL [18], respectively, which is within the linearity range of the respective drug. The data presented in Table 4 show that the applied method is suitable for the quantification of the investigated drugs in human plasma without significant interference with endogenous plasma components or with other substances.

### Application to pharmaceutical tablets

The applied method was successfully used for the quantification of FEX & PSE in their pharmaceutical combination formula, Telfast-D^®^ tablets, without any interference with the tablet matrix, which was confirmed by the results of the standard addition method (Table 3). The results of the proposed method were compared to the previously reported HPLC method in terms of the linearity and limit of detection, Table 5. In addition, a statistical comparison between the data obtained with the applied method and those obtained with the reported method [9] was performed using the t-test and the F-test. The results confirmed that no significant differences were found, as shown in Table 5.

**Table 5:** Comparison and statistical assessment of the results obtained by the proposed and the reported methods

Parameters	Proposed method		Reported method [9]	
	FEX	PSE	FEX	PSE
Linearity range	100─1500 (ng/mL)	50─1000 (ng/mL)	10–80 (µg/mL)	5–40 (µg/mL)
LOD	24.15 (ng/mL)	12.88 (ng/mL)	0.75 (µg/mL)	0.27 (µg/mL)
% R^a^	99.49	98.67	99.02	99.63
RSD	0.931	0.634	1.072	1.257
Student’s *t*-test (2.306)^b^	0.759	1.540	–	–
*F*-test (6.388)^b^	1.312	4.006	–	–

^a^ Average of five determinations

^b^ The values in parenthesis are tabulated values of “t “and “F” at (P = 0.05)

### Greenness assessment and comparison of the proposed method with reported one

Although there are many principles presented for green practices evaluation of analytical procedures, the use of national environmental index analytical eco-scale, green analytical procedure index and AGREE together were recommended for full assessment of an analytical procedure providing synergistic results. The developed spectrofluorometric method was evaluated and compared to the reported HPLC method using the mentioned tools as illustrated in Table 6. In general, national environmental index result of the spectrofluorometric method reveled the greener adherence in comparison to the reported HPLC method. Regarding to this tool did not take in consideration the amount of solvent used nor other aspects of the procedure, the analytical eco-scale score was additionally calculated, to complement national environmental index, regarded the quantities of solvents consumed and provided more information about the environmental impact of the methods. As the obtained score of the applied method was 79, this revealed an excellent green analysis method with minimal negative effect on the environment and human health. The green analytical procedure index presented a detailed overview for different steps of the analysis procedure such as sample preparation, sample handling (collection, preservation, transport & storage), chemicals used, and instrumentation. Every factor of the analytical procedures was colored green through yellow to red identifying low, medium to high negative environmental impact, respectively. The proposed method possessed the highest number of green zones and lowest number of red zones (5 green zones and one red zone). While the reported HPLC method seemed to be lower in greenness (4 green and 3 red zones). The details obtained from green analytical procedure index and the easy detection of non-eco-friendly practices makes it superior to previously mentioned methods. However, the construction of the chart is time consuming and complex. Finally, AGREE tool was applied, providing the greenness profile as a numerical value, (0.81) for spectrofluorometric method and (0.58) for the reported HPLC method, confirming the greenness superiority of the applied method. AGREE method merges the advantages and addresses the cons of the aforementioned tools. It considers the quantities of reagents, simple to construct and highlights the weaknesses of a studied method. In summary, the results obtained from all the assessment tools provided a detailed greenness profile, complemented each other, and confirmed compliance with green practices for the most part [19, 20].

**Table 6:** Greenness evaluation and comparison of the developed method and reported one using described metrics

Proposed method	Reported HPLC method [8]
National environmental method index	
	
Green analytical procedure index	
	
The AGREE evaluation method	
	

## Conclusion

In this work, a highly sensitive fully optimized first derivative synchronous spectrofluorometric method was applied for the simultaneous quantitative analysis of FEX and PSE. The described method enabled successful quantitative analysis of the studied drugs in the pure form and combined pharmaceutical tablet. Additionally, the higher sensitivity of the method provided possibility for precise quantitative analysis of FEX and PSE in spiked human plasma. The greenness of the described method was assessed using four different tools namely, the national environmental method index, the analytical eco-scale, the green analytical procedure index and The AGREE evaluation method. The proposed method seemed to be superior to the reported HPLC method with respect to the metrics of the greenness characters.

## Data Availability

The datasets used and/or analyzed during the current study are available from the corresponding author on reasonable request.

## Abbreviations

FEX: Fexofenadine hydrochloride
PSE: Pseudoephedrine hydrochloride

## References

[1] Vo-Dinh T (1978). Multicomponent analysis by synchronous luminescence spectrometry. Anal Chem.

[2] Li YQ, Li XY, Shindi AAF, Zou ZX, Liu Q, Lin LR, Li N, Geddes C (2012). Synchronous fluorescence spectroscopy and its applications in clinical analysis and food safety evaluation. Reviews in fluorescence.

[3] Attia KA, El-Olemy A, Ramzy S, Abdelazim AH, Hasan MA, Abdel-Kareem RF (2021). Simultaneous determination of elbasvir and grazoprevir in their pharmaceutical formulation by synchronous fluorescence spectroscopy coupled to dual wavelength method. Spectrochim Acta Part A.

[4] Attia KA, El-Olemy A, Ramzy S, Abdelazim AH, Hasan MA, Mohamed TF, Nasr ZA, Mohamed GF, Shahin M (2020). Development and validation of a highly sensitive second derivative synchronous fluorescence spectroscopic method for the simultaneous determination of elbasvir and grazoprevir in pharmaceutical preparation and human plasma. New J Chem.

[5] Attia KA, El-Abassawi NM, El-Olemy A, Abdelazim AH (2018). Second derivative spectrophotometric and synchronous spectrofluorometric determination of lesinurad in the presence of its oxidative degradation product. New J Chem.

[6] El-Olemy A, Abdelazim AH, Ramzy S, Hasan MA, Madkour AW, Almrasy AA, Shahin M (2021). Application of different spectrofluorimetric approaches for quantitative determination of acetylsalicylic acid and omeprazole in recently approved pharmaceutical preparation and human plasma. Spectrochim Acta Part A.

[7] Mansfield LE (2006). Once-daily immediate-release fexofenadine and sustained-release pseudoephedrine combination: a new treatment option for allergic rhinitis. Expert Opin Pharmacother.

[8] Kayesh R, Sarker ASM, Sultan M, Jahan M (2017). A simple and improved HPLC-PDA method for simultaneous estimation of fexofenadine and pseudoephedrine in extended release tablets by response surface methodology. J Chem..

[9] Karakuş S, Küçükgüzel İ, Küçükgüzel ŞG (2008). Development and validation of a rapid RP-HPLC method for the determination of cetirizine or fexofenadine with pseudoephedrine in binary pharmaceutical dosage forms. J Pharm Biomed Anal.

[10] Bharathi VD, Radharani K, Jagadeesh B, Ramulu G, Bhushan I, Naidu A, Mullangi R (2008). LC–MS–MS assay for simultaneous quantification of fexofenadine and pseudoephedrine in human plasma. Chromatographia.

[11] Mahgoub H, Gazy AA, El-Yazbi FA, El-Sayed MA, Youssef RM (2003). Spectrophotometric determination of binary mixtures of pseudoephedrine with some histamine H1-receptor antagonists using derivative ratio spectrum method. J Pharm Biomed Anal.

[12] Keith LH, Gron LU, Young JL (2007). Green analytical methodologies. Chem Rev.

[13] Gałuszka A, Migaszewski ZM, Konieczka P, Namieśnik J (2012). Analytical Eco-Scale for assessing the greenness of analytical procedures. TrAC Trends Anal Chem.

[14] Płotka-Wasylka J (2018). A new tool for the evaluation of the analytical procedure: green analytical procedure index. Talanta.

[15] Pena-Pereira F, Wojnowski W, Tobiszewski M (2020). AGREE—Analytical GREEnness metric approach and software. Anal Chem.

[16] USP C. The United States Pharmacopeia. National Formulary. 2008; 14.

[17] Branch SK (2005). Guidelines from the International Conference on Harmonisation (ICH). J Pharm Biomed Anal.

[18] Howard DR, Haribhakti R, Kittner B, Agrawala P (2005). Single-dose and steady-state bioequivalence of fexofenadine and pseudoephedrine combination tablets compared with individual formulations in healthy adults. Curr Med Res Opin.

[19] Prat D, Hayler J, Wells A (2014). A survey of solvent selection guides. Green Chem.

[20] Youssef SH, Afinjuomo F, Song Y, Garg S (2021). Development of a novel chromatographic method for concurrent determination of 5-fluorouracil and cisplatin: validation, greenness evaluation, and application on drug-eluting film. Microchem J.

